# Drivers of federal spending in pharmaceuticals of the Specialized Component: measurement and analysis

**DOI:** 10.11606/s1518-8787.2021055003097

**Published:** 2021-11-23

**Authors:** Fabiola Sulpino Vieira

**Affiliations:** I Instituto de Pesquisa Econômica Aplicada Diretoria de Estudos e Políticas Sociais Brasília DF Brasil Instituto de Pesquisa Econômica Aplicada. Diretoria de Estudos e Políticas Sociais. Brasília, DF, Brasil

**Keywords:** Drugs from the Specialized Component of Pharmaceutical Care, Drug Costs, Health Expenditures, Pharmaceutical Services, Unified Health System

## Abstract

**OBJECTIVES:**

Quantify and analyze the contribution of the main drivers of federal spending in pharmaceuticals purchase from the Specialized Component of Pharmaceutical Care (CEAF) in the period from 2010 to 2019.

**METHODS:**

An analysis of the annual expenditure's decomposition of the Brazilian Ministry of Health (MS) in pharmaceuticals from group 1A of the CEAF was carried out in order to isolate the contribution of its main drivers, price, quantity and residual, which involves therapeutic choices. This contribution's quantification was made with the support of the RStudio software version 1.3.1056 and the IndexNumR statistical package.

**RESULTS:**

The main driver of increased expenditure between 2011 and 2018 was the quantity of overlapping pharmaceuticals, 55% and 34%. In turn, the main driver in 2013 and 2015 was the residual, 33.2% and 57.9%. However, the expenditure in 2019 decreased by 30.4% compared with 2010. There was a decrease in the prices of daily treatments throughout the period. Among the years in which there was a reduction in expenditure, the residual was the main driver of the decrease in 2012 (-19.6%) and 2019 (-11.9%), while prices had the greatest impact on the decrease in expenditure in 2014 (-12%). There was also a reduction in the quantity of overlapping pharmaceuticals in three consecutive years, being -11% in 2015, -4% in 2016 and -11% in 2017. Lastly, in 2019 the reduction was -4%.

**CONCLUSIONS:**

The contribution of drivers to MS expenditure in the CEAF's 1A group fluctuated between 2010 and 2019. However, the expenditure decrease in recent years was induced by the three main drivers: price, quantity and residual. The decrease in the quantity purchased may have reduced the availability of some pharmaceuticals in the Brazilian Unified Health System (SUS).

## INTRODUCTION

Pharmaceutical spending is one of the main components of health expenditure and has concerned public administrators due to its growing trend, often higher than the rates observed for total health expenditures. This motivated several countries to implement policies to contain it, especially in recent years marked by economic and fiscal crisis^1^.

Factors such as the population's age structure, quantity consumed, prices, type of used medicines (generic or not), epidemiological profile, among others, determine expenditure on these products and are determinants of pharmaceutical spending. When these determinants induce changes in expenses between two periods, they are called expense drivers^2^.

Pharmaceutical care in the Brazilian Unified Health System (SUS) got strengthened in recent decades with the resources allocation expansion for medicines supply, and its financing was organized into three components: basic, strategic and specialized^3^. The Specialized Component of Pharmaceutical Care (CEAF) covers pharmaceuticals used in the treatment of rare and chronic diseases, generally of high cost, whose use is foreseen in lines of care contained in clinical protocols and therapeutic guidelines^4^. CEAF is divided into three groups: 1A – pharmaceuticals financed and purchased by the Brazilian Ministry of Health (MS) –; 1B – financed by the MS and by the state health departments (SES) and acquired by the SES –; and 2 – financed and acquired by the SES and municipal health departments (SMS)^5^. Expenditure on this component has a high participation in the MS's pharmaceutical budget^3^.

In the last decade, there was a reduction in the participation of the government's three spheres in the final consumption of medicines: from 10% to 8% between 2010 and 2017^6^. In addition, a 9.9% decrease in the MS's budget execution for pharmaceuticals between 2016 and 2017^7^, breaking a virtually continuous growth trajectory between 2010 and 2016^3^. The public expenditure's participation in the total expenditure on health or pharmaceuticas is an indicator related to public financing and shows the State's effort to guarantee availability and access to health goods and services by the population^8^.

Therefore, analyzes of the pharmaceutical expenditure evolution are important both for the public administration and for society, especially in countries that have not yet reached a high level of the public pharmaceutical supply, as is the case in Brazil. In the country, out-of-pocket payment is still the main access medium to these products^6^, proportionally burdening the budget of lower income households^9^. However, despite the relevance of these analyses, they are limited to a more comprehensive understanding of expenditures because they do not identify their drivers, which would help to clarify whether variations in annual expenditure are being driven by changes in prices, quantities and/or by therapeutic choices^2^.

Thus, considering this information gap, the relevance of pharmaceuticals for health care and CEAF's budgetary impact for the MS, this article aims to quantify and analyze the contribution of the main drivers of federal spending in pharmaceutical purchase from the Specialized Component of Pharmaceutical Care in the period from 2010 to 2019.

## METHODS

A decomposition analysis of the MS's annual expenditure on pharmaceuticals from CEAF's 1A group was carried out in order to isolate the contribution of its main drivers^2^. This type of analysis is based on the theory of index numbers, which establishes that the expenditure variation on a given basket of goods or services between two periods can be explained by variations in the price and quantity of goods and services that make up that basket^10^.

For pharmaceuticals, some adjustments need to be made in this theory's application, as the list offered between two periods usually varies with the exclusion or inclusion of products. Furthermore, the consumption pattern can be altered by changes in clinical practice. Thus, changes involving the quality of the medicines offered that are inadequately captured by changes in quantity are happening and, therefore, a third variable needs to be used, in addition to price and quantity, in order to explain all the variation on these products’ spending^11,12^.

Gerdtham et al^11^ called this third variable a residual. On the other hand, in later studies, other authors called it therapeutic choices^2,12,13^. The residual is influenced by changes in the pattern use of overlapping pharmaceuticals between two periods, that is, the use of medicines that are available in the two years analyzed, as well as by the exclusion and inclusion of medicines. Considering the three variables mentioned, the expenditure decomposition on pharmaceuticals can be performed as follows^11^: I*_G_*=I*_P_*.I*_Q_*.I*_R_*, where:

I*_G_* = pharmaceutical spending index (measures the real change in expenditure between two periods);

I*_P_* = price index per treatment/day (measures the effect of the real change in medication prices from an overlapping list between two periods on expenditure);

I*_Q_* = quantity index expressed in daily treatments (measures the effect of the quantities variation in an overlapping list between two periods on expenditure);

I*_R_* = residual index (measures the effect of variation in the care standard, therapeutic choices, between two periods on expenditure).

Data on pharmaceutical purchase by the MS were extracted from the Integrated System of General Services Administration (SIASG), which contains information on the goods purchased and the amounts allocated to purchases by pharmaceutical unit and the total. The data mentioned above were made available for the study to be carried out by a member of the agency's technical team.

The pharmaceuticals that are part of the CEAF list were identified, and purchases to meet legal demands were excluded, so that purchases made for SES’ distribution and subsequent dispensing in SUS units remained on the list. In SIASG, pharmaceuticals are designated by the drug name (active ingredient). Each drug on the CEAF list was further identified by its code and therapeutic subgroup of the ATC/DDD system (Anatomical Therapeutic Chemical Classification System/Defined Daily Doses), maintained by the World Health Organization (WHO), and the defined daily dose (DDD) was included for drugs that have established them^14^.

The physical quantity of each medicine in different concentrations and presentations were converted to DDD numbers per drug. Therefore, pharmaceuticals that contain the same drug are considered only one product, and their quantity is measured in DDD numbers made available in the year^11^. Lastly, only pharmaceuticals with established DDD were included in the analysis.

The amount allocated to the purchase of each medicine was divided by the number of DDDs to obtain the value or price per DDD, monetarily updated to 2019 values by the Broad Consumer Price Index (IPCA), the official inflation measurer in Brazil^15^. For pharmaceuticals with more than one purchase in the year, the average price weighted by DDD number was calculated. The annual expenditure consists of the sum of the expenditure on each drug in the year updated by the IPCA and the expenditure index was calculated considering the variation between the expenditure in one year and that carried out in the immediately preceding year.

The price and quantity indices were measured with the support of the RStudio software version 1.3.1056 and the IndexNumR package, applying the Fisher method^16-18^. Other functions of this package were used to identify the overlap of pharmaceuticals between the years analyzed and to analyze the contribution of the price and quantity variables in monetary terms to the annual variation in expenditure^18^. Lastly, the residual index was calculated using the formula proposed by Gerdtham et al^11^, where: I*_R_*=I*_G_*/(I*_P_*.I*_Q_*).

## RESULTS

**Figure 1:**
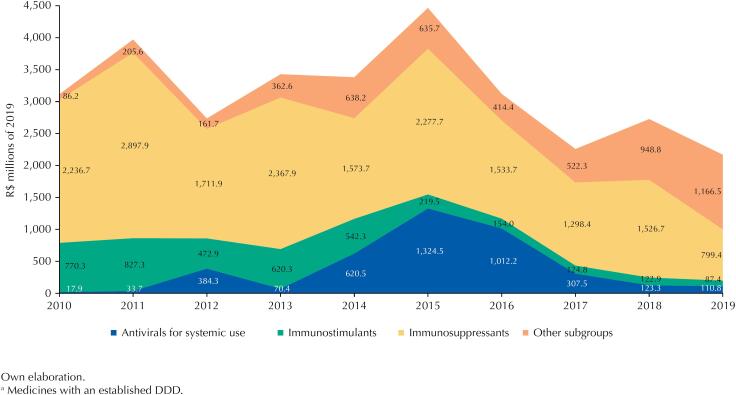
Expenditure (in millions of R$ in 2019) of the Brazilian Ministry of Health on pharmaceuticals of the CEAF's 1A group^a^ by therapeutic subgroups (2010–2019).

Source: SIASG.

Between 2010 and 2019, the MS acquired 83 different drugs that were or are part of CEAF's 1A group. Of this total, 20 drugs (24.1%) do not have a DDD established by the WHO and totaled R$ 3.77 billion in 2019 values in the period spent. The other drugs (n = 63; 75.9%) have an established DDD and compose the pharmaceuticals list analyzed in this study, totaling expenditure of R$ 31.31 billion. Figure 1 shows the annual expenditure of the MS in this list, according to the main therapeutic subgroups in terms of spending.

Immunosuppressants constitute the subgroup with the highest expenditure until 2018. In 2019, the expenses of this subgroup lose the first position for products that integrate other therapeutic subgroups, meaning a reduction of 47.6% compared with 2018. Also highlighted in this Figure is the growth in expenditure on immunosuppressants and antivirals for systemic use between 2014 and 2015, the successive reductions in expenses with these subgroups in 2016 and 2017 and the increase in expenses with other therapeutic subgroups in 2018 and 2019.

Table 1 shows the result of the overlapping analysis of pharmaceuticals purchased in two periods (overlapping medication list), comparing purchases made in one year in relation to the immediately preceding year. Of the 17 drugs purchased in 2011, 12 had been purchased in 2010, representing an overlap of 70.6% of drugs. In terms of expenditure, out of the total expenditure carried out in 2011 (R$ 3.96 billion), R$ 3.57 billion were allocated to the purchase of these 12 drugs, which is equivalent to 89.9% of that year's expenditure.

**Table 1:** Drugs purchased and pharmaceutical expenditure from CEAF's^a^ 1A group by the Brazilian Ministry of Health in the current year and in the previous year (2010–2019).

Year	Drugs			Expenditure (in R$ of 2019)		
	Purchased in the year (A)	Purchased in the current year and the previous year (overlapping) (B)	Frequency in % (C) = (B)/(A)	Total in the year (D)	In drugs purchased in the current year and in the previous year (overlapping) (E)	Frequency in % (F) = (E)/(D)
2010	13	–	–	3,111,101,607	–	–
2011	17	12	70.6	3,964,405,181	3,565,115,413	89.9
2012	13	9	69.2	2,730,880,595	2,224,139,542	81.4
2013	20	9	45.0	3,421,253,546	2,203,442,115	64.4
2014	26	16	61.5	3,374,752,748	2,403,345,441	71.2
2015	30	20	66.7	4,457,392,520	2,143,408,273	48.1
2016	27	23	85.2	3,114,255,832	2,834,934,548	91.0
2017	30	21	70.0	2,253,005,743	1,848,589,843	82.0
2018	36	21	58.3	2,721,660,204	1,866,013,403	68.6
2019	41	25	61.0	2,164,179,182	1,509,499,956	69.7

Source: SIASG.

Own elaboration.

a Medicines with an established DDD.

The year in which there was the greatest overlap in drug purchases was 2016 compared with 2015 (85.2%), whose expenditure on overlapping pharmaceuticals was 91.0% (R$ 2.83 billion out of R$ 3.11 billion). The smallest overlaps of drugs purchased compared with the previous year occurred in 2013 (45.0%) and 2018 (58.3%), with expenditures on overlapping pharmaceuticals of R$ 2.2 billion (64.4% of R$ 3.4 billion) and R$ 1.7 billion (68.6% of R$ 1.9 billion) respectively. As for expenditure on overlapping items, a significant reduction is still present (-57.7%) in the comparison between the amount allocated in 2011 (R$ 3.57 billion) and the amount allocated in 2019 (R$ 1.51 billion).

Table 2 shows the portion of the expenditure allocated to the purchase of overlapping pharmaceuticals and the one allocated to the purchase of non-overlapping ones. While expenditure on overlapping drugs showed a decreasing tendency, expenditure on non-overlapping drugs varied significantly, registering the highest value in 2015, when it reached the level of R$ 2.31 billion, representing 51.9% of the annual expenditure. The lowest expenses with non-overlapping pharmaceuticals occurred in 2016 (R$ 279.32 million) and in 2011 (R$ 399.29 million).

**Table 2:** Brazilian Ministry of Health's expenditure on pharmaceuticals of CEAF's^a^ 1A group, according to a list of overlapping pharmaceuticals between the current year and the previous year, and a list of non-overlapping pharmaceuticals (2010–2019).

Year	Expenditure on the year in R$ of 2019 (A)	Expenditure on overlapping pharmaceuticals		Expenditure on non-overlapping pharmaceuticals	
		Value in R$ of 2019 (B)	Frequency in % (C) = (B)/(A)	Value in R$ of 2019 (D)	Frequency in % (E) = (D)/(A)
2010	3,111,101,607	–	–	–	–
2011	3,964,405,181	3,565,115,413	89.9	399,289,768	10.1
2012	2,730,880,595	2,224,139,542	81.4	506,741,053	18.6
2013	3,421,253,546	2,203,442,115	64.4	1,217,811,431	35.6
2014	3,374,752,748	2,403,345,441	71.2	971,407,307	28.8
2015	4,457,392,520	2,143,408,273	48.1	2,313,984,247	51.9
2016	3,114,255,832	2,834,934,548	91.0	279,321,284	9.0
2017	2,253,005,743	1,848,589,843	82.0	404,415,900	18.0
2018	2,721,660,204	1,866,013,403	68.6	855,646,801	31.4
2019	2,164,179,182	1,509,499,956	69.7	654,679,226	30.3

Source: SIASG.

Own elaboration.

a Medicines with an established DDD.

**Figure 2:**
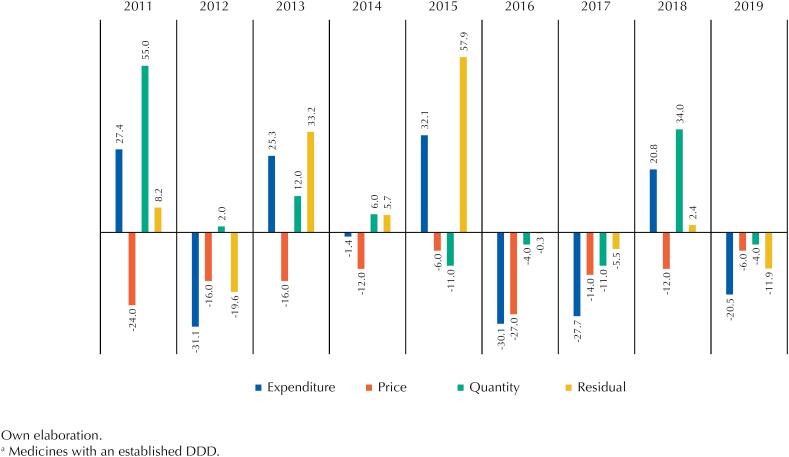
Annual variation (in relation to the immediately preceding year - %) of expenditure on pharmaceuticals of CEAF's^a^ 1A group and its drivers (2011–2019).

Source: SIASG.

The annual variation of the pharmaceuticals expenditure's main drivers in CEAF's 1A group is shown in Figure 2. As shown, expenditure was reduced compared with the previous year in 2012 (-31.1%), 2014 (-1.4%), 2016 (-30.1%), 2017 (-27.7%) and 2019 (-20.5%), and increased in 2011 (27.4%), 2013 (25.3%), 2015 (32.1%) and 2018 (20.8%). The prices of overlapping pharmaceuticals decreased to a greater or lesser extent throughout the period. In turn, the quantity of these purchased medications varied. In 2011 and 2018, the main driver of the increase in annual expenditure was the quantity of overlapping items compared with the previous year, 55% and 34% respectively. In 2013 and 2015, the main driver was the residual, 33.2% and 57.9% respectively.

In the years that had a reduction in expenditure, the residual was the main driver of the reduction in 2012 (-19.6%) and 2019 (-11.9%), while prices had the greatest impact on the decrease in expenditure in 2014 (-12%). We highlight the reductions in the quantity of overlapping items in three consecutive years, 2015 (-11%), 2016 (-4%) and 2017 (-11%), and in 2019 (-4%). Over the past three years, the main drivers of expenditure, price, quantity and residual, have contributed to the decrease in expenses on pharmaceuticals, however, the reduction in quantity draws more attention.

In Table 3 it is possible to observe the contribution of drivers, price and quantity to the annual variation of expenditure in monetary terms. The decrease in the prices of overlapping items between 2010 and 2011 generated savings of R$ 942.75 million. However, the increase in the quantity purchased of these items generated an additional expense of R$ 1.48 billion, which caused the expenses on overlapping items to produce a balance of R$ 540.20 million. Expenditure on overlapping items between 2010 and 2011 was R$ 3.57 billion and expenditure on non-overlapping pharmaceuticals contributed R$ 399.29 million, totaling annual expenditure of R$ 3.96 billion in 2011.

**Table 3:** Expenditure decomposition of the Brazilian Ministry of Health on pharmaceuticals of CEAF's^a^ 1A group, for the variables price and quantity of overlapping pharmaceuticals (2011–2019). (In R$ of 2019)

Year	Expenditure in the year (A)	Overlapping pharmaceuticals^b^ (purchased in the current year and in the previous year)				Expenditure on non-overlapping pharmaceuticals (F) = (A) - (E)
		Price (B)	Quantity (C)	Change (D) = (B) + (C)	Expenditure (E)	
2010	3,111,101,607	–	–	–	–	–
2011	3,964,405,181	−942,747,326	1,482,949,631	540,202,305	3,565,115,413	399,289,768
2012	2,730,880,595	−429,401,035	55,202,768	−374,198,267	2,224,139,542	506,741,053
2013	3,421,253,546	−409,206,701	262,862,794	−146,343,906	2,203,442,115	1,217,811,431
2014	3,374,752,748	−319,312,207	157,940,963	−161,371,244	2,403,345,441	971,407,307
2015	4,457,392,520	−152,411,230	−270,527,006	−422,938,236	2,143,408,273	2,313,984,247
2016	3,114,255,832	−1,065,511,265	−139,082,756	−1,204,594,021	2,834,934,548	279,321,284
2017	2,253,005,743	−317,851,837	−240,357,431	−558,209,268	1,848,589,843	404,415,900
2018	2,721,660,204	−214,323,069	503,440,831	289,117,762	1,866,013,403	855,646,801
2019	2,164,179,182	−104,657,061	−62,468,364	−167,125,425	1,509,499,956	654,679,226

Source: SIASG.

Own elaboration.

a Medicines with an established DDD.

b It is not possible to separate the residual's contribution arising from any changes in the pattern of use of overlapping pharmaceuticals between two years in this analysis, as the expenditure (E) refers to the expenditure with the overlapping list in the two compared periods, this list being different in the analyzed series, as can be seen in the column of the number of overlapping drugs in Table 1 of this article.

c The residual involves expenses arising from the purchase of non-overlapping pharmaceuticals and those arising from changes in the use pattern of overlapping pharmaceuticals. In this column, expenditures in the current year related to the contribution of non-overlapping pharmaceuticals are presented.

Furthermore, it can be seen in Table 3 that, due to the reduction in prices, the biggest savings occurred in 2011 and 2016. Reduced values also occurred due to the decrease in quantities in 2015 (R$ 270.53 million), 2016 (R$ 139.08 million), 2017 (R$ 240.36 million) and 2019 (R$ 62.47 million).

## DISCUSSION

The results show a great impact of some therapeutic subgroups on the expenditure of the MS. In almost every year, immunosuppressants were the biggest expense. In an analysis that considered all purchases of pharmaceuticals from this subgroup by federal bodies, it was identified that the main driver of the spending was the amount purchased in the period of 2010 to 2015^19^. The increase on the expenditure on immunosuppressants is related to the increase in organ and bone marrow transplants in Brazil^20^, with the expansion of access and with a possible increase in autoimmune diseases^21^. The reduction in expenditure identified in 2019 compared with 2018, considering that the number of transplants has continued to grow in recent years^20^, may be related to the reduction in the availability of these medicines for the treatment of autoimmune diseases and the reduction in the prices of these products in general.

It is noteworthy the growth of expenditure on antivirals for systemic use used in the treatment of hepatitis B and C between 2014 and 2016 and its reduction as of 2017. During this period, there were changes in the pattern of treatment for hepatitis C. In 2015, three medications were incorporated: sofosbuvir, daclatasvir and simeprevir^22^. Data from 2016 shows that there was a patent granted for simeprevir and that, although the other two drugs were not protected by patents, they were exclusively marketed in Brazil by transnational pharmaceutical companies^23^. The exclusivity of supply in both cases usually leads to the offer of products at high prices. Also in 2016, the National Commission for Technology Incorporation at SUS (Conitec) recommended the exclusion of the medications telaprevir and boceprevir^24^. Furthermore, several antivirals used in the treatment of hepatitis C were objects of Partnerships for Productive Development and this may have contributed to the reduction of product prices over time^23^. These events help to explain the observed movement of expenditure in this therapeutic subgroup.

As for the number of drugs, there was an expansion of the list acquired centrally by the MS, as shown in Table 1. Two situations justify this increase. The first is the agreement between SUS managers so that the federal government assumes the acquisition of pharmaceuticals already incorporated into the system^4^. The second situation is the incorporation of medicines, which increased with the institution of Conitec in 2011^25^ and which have already entered SUS under the purchase responsibility by the MS^26^. A joint analysis of data from the Ambulatory Care Information System (SIA) and CEAF's pharmaceuticals lists reveals that between 2012 and 2018 the balance of incorporations and disembodiments in 1A group was 17 drugs and that centralized purchase in the MS increased by 26 drugs (from 30 to 56)^27^.

It is also interesting to note in Table 1 the percentage of overlapping of the purchased drugs’ list between two consecutive years. As in no year there was an overlap of 100% and the number of drugs purchased increased in most years, these facts indicate centralization, incorporation and purchase of an item that was already under the responsibility of the MS that was not purchased in the previous year. In addition, as drug overlap occurs in a smaller number in the current year compared with the total of drugs purchased in the previous year, evidence that purchase rarely occurs annually for several drugs exists, as it is unlikely that most of them are no longer purchased for having been excluded from SUS^28^ and for the occurrence of drug transfer from 1A group to 1B group^26^. For example, in 2019, 25 drugs were purchased that were also purchased in 2018. But the total of drugs purchased in 2018 was 36, so 11 drugs purchased in 2018 were not purchased in 2019.

Expenditure on non-overlapping drugs between consecutive years was particularly high in 2015 (R$ 2.3 billion), as shown in Table 2. This fact is associated with decisions to centralize the purchase and the incorporation of medicines between 2014 and 2015. After the incorporation decision, SUS has up to 180 days to make the pharmaceuticals available in its pharmacies^29^. Another element that stands out in this table is the reduction in expenditure on the group of overlapping pharmaceuticals from 2016 and on non-overlapping pharmaceuticals in 2016 compared with 2015. In the first group, the variation of expenses between 2016 and 2019 was -46.8% and, in the second group, -71.7% between 2015 and 2019. Obviously, the medicines list in the second group differs each year and the centralization or incorporation of more or less expensive products can influence expenditure. However, it cannot be ruled out that the reduction is the result of budgetary restrictions, aggravated by the approval of the Expenditure Ceiling Amendment in 2016 (EC 95). Between 2016 and 2017, the expenses of the MS with CEAF decreased by 21.7%, from R$ 6.9 billion to R$ 5.4 billion at 2017 prices^7^.

More worrying is the reduction in the first group. If between 2010 and 2019 there was an increase in the drugs purchased by the MS, either through centralization or incorporation, an increase in expenditure was to be expected, unless the prices of the products significantly reduced. Furthermore, if the quantity of daily treatments per therapeutic subgroup was constant, there would be an indication that the new medications replaced the older ones. In fact, prices decreased in all the years observed, as shown in Figure 2, because of increasing the purchasing power of the MS due to its capacity to negotiate with exclusive suppliers and the scale of acquisition in the bids. However, the quantity of daily treatments made available also reduced from 2015 to 2017 and in 2019 compared with the previous year. Table 3 makes the effect of the decrease in quantity on expenditure clear in these years.

In summary, MS expenditure on the purchase of CEAF's pharmaceuticals that have an established DDD fluctuated in the period from 2010 to 2019, with a significant increase in the middle of that decade. However, it registered a decrease of 30.4% in the comparison between 2019 in relation to 2010, despite the purchase's centralization and the medicines’ incorporation having supplanted the exclusion or the transfer of responsibility for the purchase to the SES^27^. The reduction in the prices of daily treatments with overlapping drugs for two consecutive years contributed to this reduction throughout the period. Nevertheless, there was also a reduction in the purchase of daily treatments in recent years, with a probable impairment of availability in SUS for those who had reduced the quantity, considering the reports of lack of CEAF's pharmaceuticals under the responsibility of the MS in SUS^30,31^. The increase in residual in two years of the analyzed series was the main driver of the increase in expenditure in those years, which is mainly due to the centralization of purchase and incorporation of medications.

This study has, of course, some limitations. The first is that the methodology used makes it impossible to analyze pharmaceuticals that do not have an established DDD and, therefore, 10.8% of the expenditure on CEAF's 1A group was not analyzed. The second is that, since the database used only includes public procurement data, only the effects of the main drivers on expenditure were estimated. With the use of complementary data, for example, from the Authorization for High-Complexity Procedures (APACs) and from medicine registrations, it is possible to decompose the main indexes into other indexes, measuring the contribution of drivers such as the purchase of generics, volume and extension of prescriptions, among others, that impact the main drivers^2,32^. Lastly, the third limitation involves information on the quantity purchased and, consequently, on the total value, as, in the methodology applied in this study, the cost of each medication is calculated from the multiplication of the unit price and the quantity negotiated or expected in the purchase, contained in the SIASG. The annual expenditure is the sum of the expenses with pharmaceuticals purchased in the year. Although the Brazilian Federal Government Purchasing Panel contains information on the budget-financial execution of the MS by contract, information on the expense committed, settled and paid per medication item with the respective quantity was not disclosed. Therefore, we worked with the amount foreseen in the purchase.

However, despite these limitations, the study makes a significant contribution to the understanding of the main factors that impact most of the MS's expenditure in the CEAF's 1A group. The analysis of the drivers of pharmaceutical expenditure proves to be a useful tool for the management of pharmaceutical care, as it produces information that enables a broader understanding of the behavior of variables that impact expenditure and can guide the adoption of measures aimed at guaranteeing the population's access to medicines and the rational use of financial resources.
