# Supplementary material for: Drivers of federal spending in pharmaceuticals of the Specialized Component: measurement and analysis
Source: Rev Saude Publica. 2021 Nov 23;55:91. doi: 10.11606/s1518-8787.2021055003097 (PMC8647991; doi:10.11606/s1518-8787.2021055003097)
Supplement: Translation

# Translation: Indutores do gasto federal em medicamentos do componente especializado: medição e análise

**Authors:** Fabiola Sulpino Vieira

**Affiliations:** I Brasília DF Brasil Instituto de Pesquisa Econômica Aplicada. Diretoria de Estudos e Políticas Sociais. Brasília, DF, Brasil

**Keywords:** Medicamentos do Componente Especializado da Assistência Farmacêutica, Custos de Medicamentos, Gastos em Saúde, Assistência Farmacêutica, Sistema Único de Saúde

## Abstract

**OBJETIVOS:**

Quantificar e analisar a contribuição dos indutores principais do gasto federal na aquisição de medicamentos do Componente Especializado da Assistência Farmacêutica (CEAF) no período de 2010 a 2019.

**MÉTODOS:**

Realizou-se análise de decomposição do gasto anual do Ministério da Saúde (MS) em medicamentos do grupo 1A do CEAF a fim de isolar a contribuição dos seus indutores principais, preço, quantidade e resíduo, que envolve as escolhas terapêuticas. A quantificação dessa contribuição foi feita com o suporte do software RStudio versão 1.3.1056 e do pacote estatístico IndexNumR.

**RESULTADOS:**

O principal indutor do aumento do gasto entre 2011 e 2018 foi a quantidade dos medicamentos sobrepostos, 55% e 34%. Por sua vez, o indutor principal em 2013 e 2015 foi o resíduo, 33,2% e 57,9%. Entretanto, o gasto em 2019 registrou queda de 30,4% em relação a 2010. Houve diminuição dos preços dos tratamentos diários em todo o período. Entre os anos em que houve redução do gasto, o resíduo foi o principal indutor da queda em 2012 (-19,6%) e 2019 (-11,9%), enquanto os preços tiveram maior impacto na diminuição do gasto em 2014 (-12%). Houve ainda redução da quantidade dos medicamentos sobrepostos em três anos consecutivos, sendo -11% em 2015, -4% em 2016 e -11% em 2017. Por fim, em 2019 a redução foi de -4%.

**CONCLUSÕES:**

A contribuição dos indutores para o gasto do MS no grupo 1A do CEAF oscilou entre 2010 e 2019. Entretanto, a queda do gasto em anos recentes foi induzida pelos três indutores principais: preço, quantidade e resíduo. A diminuição da quantidade adquirida pode ter reduzido a disponibilidade de alguns medicamentos no Sistema Único de Saúde (SUS).

## INTRODUÇÃO

O gasto em medicamentos constitui um dos principais componentes do gasto em saúde e tem preocupado gestores públicos por sua tendência de crescimento, muitas vezes superiores às taxas observadas para as despesas totais em saúde. Isto motivou diversos países a implementar políticas para as suas contenções, especialmente em anos recentes marcados por crise econômica e fiscal^1^.

Fatores como a estrutura etária da população, quantidade consumida, preços, tipo de medicamentos utilizados (genéricos ou não), perfil epidemiológico, entre outros, determinam as despesas nesses produtos e são designados determinantes do gasto em medicamentos. Quando esses determinantes induzem alterações das despesas entre dois períodos, eles são chamados de indutores do gasto^2^.

No Brasil, a assistência farmacêutica no Sistema Único de Saúde (SUS) se fortaleceu nas últimas décadas com a ampliação da alocação de recursos para a oferta de medicamentos, tendo sido o seu financiamento organizado em três componentes: básico, estratégico e especializado^3^. O Componente Especializado da Assistência Farmacêutica (CEAF) é o que abrange medicamentos usados no tratamento de doenças raras e crônicas, geralmente de alto custo, cujo uso está previsto em linhas de cuidado constantes de protocolos clínicos e diretrizes terapêuticas^4^. O CEAF está dividido em três grupos: 1A – medicamentos financiados e adquiridos pelo Ministério da Saúde (MS) –; 1B – financiados pelo MS e pelas secretarias estaduais de saúde (SES), e adquiridos pelas SES –; e 2 – financiados e adquiridos pelas SES e secretarias municipais de saúde (SMS)^5^. As despesas com esse componente têm elevada participação no orçamento de medicamentos do MS^3^.

Na última década, houve redução da participação das três esferas de governo nas despesas de consumo final de medicamentos: de 10% para 8% entre 2010 e 2017^6^. Além disso, diminuição de 9,9% da execução orçamentária do MS para medicamentos entre 2016 e 2017^7^, rompendo com uma trajetória de crescimento praticamente contínua entre 2010 e 2016^3^. A participação da despesa pública na despesa total em saúde ou em medicamentos é um indicador que se relaciona com o financiamento público e que evidencia o esforço do Estado para a garantia da disponibilidade e do acesso a bens e a serviços de saúde pela população^8^.

Por isso, análises sobre a evolução do gasto em medicamentos são importantes tanto para a administração pública quanto para a sociedade, especialmente em países que ainda não alcançaram elevado patamar de oferta pública de medicamentos como é o caso do Brasil. No país, o pagamento direto do bolso (*out-of-pocket*) ainda constitui o principal meio para acesso a esses produtos^6^, onerando proporcionalmente mais o orçamento das famílias de menor renda^9^. Contudo, a despeito da relevância dessas análises, elas são limitadas para uma compreensão mais abrangente sobre as despesas porque não identificam os seus indutores, o que ajudaria a esclarecer se variações do gasto anual estão sendo dirigidas por mudanças nos preços, nas quantidades e/ou pelas escolhas terapêuticas^2^.

Assim, considerando essa lacuna de informação, a relevância dos medicamentos para a atenção à saúde e o impacto orçamentário do CEAF para o MS, este artigo tem por objetivos quantificar e analisar a contribuição dos indutores principais do gasto federal na aquisição de medicamentos do Componente Especializado da Assistência Farmacêutica no período de 2010 a 2019.

## MÉTODOS

Realizou-se análise de decomposição do gasto anual do MS em medicamentos do grupo 1A do CEAF a fim de isolar a contribuição dos seus principais indutores^2^. Esse tipo de análise baseia-se na teoria dos números-índices, que estabelece que a variação do gasto com determinada cesta de bens ou serviços entre dois períodos pode ser explicada por variações no preço e na quantidade dos bens e serviços que compõem essa cesta^10^.

Para os medicamentos, alguns ajustes precisam ser feitos na aplicação dessa teoria, pois a lista ofertada entre dois períodos geralmente sofre variação com a exclusão ou inclusão de produtos. Além disso, o padrão de consumo pode ser alterado por mudanças na prática clínica. Com isso, há alterações envolvendo a qualidade dos medicamentos ofertados que não são adequadamente captadas por mudanças na quantidade e, portanto, uma terceira variável precisa ser utilizada, para além do preço e da quantidade, a fim de explicar toda a variação do gasto nesses produtos^11,12^.

Gerdtham et al^11^ chamaram essa terceira variável de resíduo. Por sua vez, em trabalhos posteriores, outros autores a denominaram de escolhas terapêuticas^2,12,13^. O resíduo é influenciado por alterações no padrão de uso de medicamentos sobrepostos entre dois períodos, ou seja, na utilização de medicamentos que estão disponíveis nos dois anos analisados, assim como pela exclusão e inclusão de medicamentos. Considerando as três variáveis mencionadas, a decomposição do gasto em medicamentos pode ser realizada como segue^11^: I*_G_*=I*_P_*.I*_Q_*.I*_R_*, onde:

I*_G_* = índice do gasto em medicamentos (mede a variação real do gasto entre dois períodos);

I*_P_* = índice dos preços por tratamento/dia (mede o efeito da variação real dos preços dos medicamentos de uma lista sobreposta entre dois períodos sobre o gasto);

I*_Q_* = índice das quantidades expressas em tratamentos diários (mede o efeito da variação das quantidades de uma lista sobreposta entre dois períodos sobre o gasto);

I*_R_* = índice dos resíduos (mede o efeito da variação do padrão de tratamento, escolhas terapêuticas, entre dois períodos sobre o gasto).

Dados da aquisição de medicamentos pelo MS foram extraídos do Sistema Integrado de Administração de Serviços Gerais (SIASG), que contêm informações sobre os bens comprados e os valores alocados às aquisições por unidade farmacotécnica e total. Os dados mencionados acima foram disponibilizados para a realização do estudo por integrante da equipe técnica do próprio órgão.

Os medicamentos que fazem parte da lista do CEAF foram identificados, sendo excluídas as compras para atendimento a demandas judiciais, de forma que restaram na lista as aquisições feitas para distribuição às SES e posterior dispensação nas unidades do SUS. No SIASG, os medicamentos são designados pelo nome do fármaco (princípio-ativo). Cada fármaco da lista do CEAF foi adicionalmente identificado pelo seu código e subgrupo terapêutico do sistema ATC/DDD *(Anatomical Therapeutic Chemical Classification System/ Defined Daily Doses)*, mantido pela Organização Mundial da Saúde (OMS), e a dose diária definida (DDD) foi incluída para os medicamentos que as têm estabelecida^14^.

As quantidades físicas de cada medicamento em distintas concentrações e apresentações foram convertidas para números de DDD por fármaco. Dessa forma, os medicamentos que contêm o mesmo fármaco são considerados apenas um produto, sendo sua quantidade medida em números de DDD disponibilizadas no ano^11^. Por fim, foram incluídos na análise apenas os medicamentos com DDD estabelecida.

O valor alocado à compra de cada fármaco foi dividido pelo número de DDDs para obtenção do valor ou preço por DDD, atualizados monetariamente para valores de 2019 pelo Índice de Preços ao Consumidor Amplo (IPCA), medidor oficial de inflação no Brasil^15^. Para os medicamentos com mais de uma aquisição no ano, calculou-se o preço médio ponderado por número de DDD. O gasto anual consiste na soma do gasto com cada fármaco no ano corrigido pelo IPCA e o índice do gasto foi calculado considerando a variação entre o gasto de um ano e o realizado no ano imediatamente anterior.

Os índices dos preços e das quantidades foram mensurados com o suporte do *software RStudio* versão 1.3.1056 e do pacote *IndexNumR*, aplicando-se o método de Fisher^16-18^. Outras funções desse pacote foram utilizadas para identificação da sobreposição dos medicamentos entre os anos analisados e análise da contribuição das variáveis preço e quantidade em termos monetários para a variação anual do gasto^18^. Por fim, o índice do resíduo foi calculado utilizando-se a fórmula proposta por Gerdtham et al^11^, onde: I_R_=I_G_/(I_P_.I_Q_).

## RESULTADOS

Entre 2010 e 2019, o MS adquiriu 83 fármacos distintos que integraram ou integram o grupo 1A do CEAF. Deste total, 20 fármacos (24,1%) não têm DDD estabelecida pela OMS e somaram no período gasto de R$ 3,77 bilhões em valores de 2019. Os demais fármacos (n = 63–75,9%) têm DDD estabelecida e compuseram a lista de medicamentos analisada neste estudo, totalizando gasto de R$ 31,31 bilhões. A Figura 1 apresenta o gasto anual do MS nessa lista, segundo os principais subgrupos terapêuticos em termos de despesa.

**Figura 1:** Gasto (em milhões de R$ de 2019) do Ministério da Saúde em medicamentos do grupo 1A do CEAF^a^ por subgrupos terapêuticos (2010–2019).

Fonte: SIASG.

Verifica-se que os imunossupressores constituem o subgrupo de maior gasto até 2018. Em 2019, as despesas deste subgrupo perdem a primeira posição para produtos que integram outros subgrupos terapêuticos, significando uma redução de 47,6% em relação a 2018. Destacam-se ainda nessa Figura o crescimento do gasto em imunossupressores e em antivirais para uso sistêmico entre 2014 e 2015, as sucessivas reduções das despesas com esses subgrupos em 2016 e 2017 e o aumento das despesas com outros subgrupos terapêuticos em 2018 e 2019.

Na Tabela 1, apresenta-se o resultado da análise de sobreposição dos medicamentos comprados em dois períodos (lista sobreposta de medicamentos), comparando-se as aquisições realizadas em um ano em relação ao ano imediatamente anterior. Observa-se que dos 17 fármacos adquiridos em 2011, 12 tinham sido comprados em 2010, representando uma sobreposição de 70,6% dos fármacos. Em termos de gasto, da despesa total realizada em 2011 (R$ 3,96 bilhões), R$ 3,57 bilhões foram alocados à aquisição desses 12 fármacos, o que equivale a 89,9% do gasto daquele ano.

**Tabela 1:** Fármacos comprados e gasto realizado em medicamentos do grupo 1A do CEAF^a^ pelo Ministério da Saúde no ano corrente e no ano anterior (2010–2019).

Ano	Fármacos			Gasto (em R$ de 2019)		
	Comprados no ano (A)	Comprados no ano corrente e no ano anterior (sobrepostos) (B)	Frequência em % (C) = (B)/(A)	Total no ano (D)	Em fármacos comprados no ano corrente e no ano anterior (sobrepostos) (E)	Frequência em % (F) = (E)/(D)
2010	13	–	–	3.111.101.607	–	–
2011	17	12	70,6	3.964.405.181	3.565.115.413	89,9
2012	13	9	69,2	2.730.880.595	2.224.139.542	81,4
2013	20	9	45,0	3.421.253.546	2.203.442.115	64,4
2014	26	16	61,5	3.374.752.748	2.403.345.441	71,2
2015	30	20	66,7	4.457.392.520	2.143.408.273	48,1
2016	27	23	85,2	3.114.255.832	2.834.934.548	91,0
2017	30	21	70,0	2.253.005.743	1.848.589.843	82,0
2018	36	21	58,3	2.721.660.204	1.866.013.403	68,6
2019	41	25	61,0	2.164.179.182	1.509.499.956	69,7

Fonte: SIASG.

Elaboração própria.

a Medicamentos com DDD estabelecida.

O ano em que houve maior sobreposição da compra de fármacos foi 2016 em relação a 2015 (85,2%), cujo gasto nos medicamentos sobrepostos foi de 91,0% (R$ 2,83 bilhões dos R$ 3,11 bilhões). As menores sobreposições dos fármacos adquiridos em relação ao ano anterior ocorreram em 2013 (45,0%) e 2018 (58,3%), com gastos nos medicamentos sobrepostos de R$ 2,2 bilhões (64,4% de R$ 3,4 bilhões) e R$ 1,7 bilhão (68,6% de R$ 1,9 bilhão) respectivamente. Quanto ao gasto em itens sobrepostos, verifica-se ainda redução significativa (-57,7%) na comparação entre o valor alocado em 2011 (R$ 3,57 bilhões) e o alocado em 2019 (R$ 1,51 bilhão).

A Tabela 2 explicita a parcela do gasto que foi alocada à aquisição dos medicamentos sobrepostos e a que foi destinada à compra dos não sobrepostos. Nota-se que, enquanto a despesa com fármacos sobrepostos apresentou tendência de redução, a despesa com os não sobrepostos variou significativamente, registrando o maior valor em 2015, quando atingiu o patamar de R$ 2,31 bilhões, representando 51,9% do gasto anual. As menores despesas com medicamentos não sobrepostos ocorreram em 2016 (R$ 279,32 milhões) e em 2011 (R$ 399,29 milhões).

**Tabela 2:** Gasto do Ministério da Saúde em medicamentos do grupo 1A do CEAF^a^, segundo uma lista sobreposta de medicamentos entre o ano corrente e o ano anterior e uma lista de medicamentos não sobrepostos (2010–2019).

Ano	Gasto no ano em R$ de 2019 (A)	Gasto em medicamentos sobrepostos		Gasto em medicamentos não sobrepostos	
		Valor em R$ de 2019 (B)	Frequência em % (C) = (B)/(A)	Valor em R$ de 2019 (D)	Frequência em % (E) = (D)/(A)
2010	3.111.101.607	–	–	–	–
2011	3.964.405.181	3.565.115.413	89,9	399.289.768	10,1
2012	2.730.880.595	2.224.139.542	81,4	506.741.053	18,6
2013	3.421.253.546	2.203.442.115	64,4	1.217.811.431	35,6
2014	3.374.752.748	2.403.345.441	71,2	971.407.307	28,8
2015	4.457.392.520	2.143.408.273	48,1	2.313.984.247	51,9
2016	3.114.255.832	2.834.934.548	91,0	279.321.284	9,0
2017	2.253.005.743	1.848.589.843	82,0	404.415.900	18,0
2018	2.721.660.204	1.866.013.403	68,6	855.646.801	31,4
2019	2.164.179.182	1.509.499.956	69,7	654.679.226	30,3

Fonte: SIASG.

Elaboração própria.

a Medicamentos com DDD estabelecida.

A variação anual dos principais indutores do gasto em medicamentos do grupo 1A do CEAF é apresentada na Figura 2. Conforme pode ser demonstrado, o gasto sofreu reduções em relação ao ano anterior em 2012 (-31,1%), 2014 (-1,4%), 2016 (-30,1%), 2017 (-27,7%) e 2019 (-20,5%), e crescimento em 2011 (27,4%), 2013 (25,3%), 2015 (32,1%) e 2018 (20,8%). Observa-se que os preços dos medicamentos sobrepostos decresceram em maior ou menor magnitude em todo o período. Por sua vez, a quantidade adquirida desses medicamentos apresentou variação. Em 2011 e 2018, o principal indutor do aumento do gasto anual foi a quantidade dos itens sobrepostos em relação ao ano anterior, 55% e 34% respectivamente. Já em 2013 e 2015, o indutor principal foi o resíduo, 33,2% e 57,9% respectivamente.

**Figura 2:** Variação anual (em relação ao ano imediatamente anterior - %) do gasto em medicamentos do grupo 1A do CEAF^a^ e de seus indutores (2011–2019).

Fonte: SIASG.

Entre os anos em que houve redução do gasto, o resíduo foi o principal indutor da queda em 2012 (-19,6%) e 2019 (-11,9%), enquanto os preços tiveram maior impacto na diminuição do gasto em 2014 (-12%). Destacam-se as reduções de quantidade dos itens sobrepostos em três anos consecutivos, 2015 (-11%), 2016 (-4%) e 2017 (-11%), e em 2019 (-4%). Nesses três últimos anos, os indutores principais do gasto, preço, quantidade e resíduo, contribuíram para a diminuição das despesas em medicamentos, contudo, a queda da quantidade chama mais a atenção.

Na Tabela 3 é possível observar a contribuição dos indutores, preço e quantidade para a variação anual do gasto em termos monetários. Observa-se que a diminuição dos preços dos itens sobrepostos entre 2010 e 2011 gerou uma economia de R$ 942,75 milhões. Entretanto, o aumento da quantidade adquirida desses itens gerou gasto adicional de R$ 1,48 bilhão, o que fez com que a despesas nos itens sobrepostos produzisse um saldo de R$ 540,20 milhões. O gasto com os itens sobrepostos entre 2010 e 2011 foi de R$ 3,57 bilhões e o gasto com medicamentos não sobrepostos contribuiu com R$ 399,29 milhões, totalizando o gasto anual de R$ 3,96 bilhões em 2011.

**Tabela 3:** Decomposição do gasto do Ministério da Saúde em medicamentos do grupo 1A do CEAF^a^, para as variáveis preço e quantidade dos medicamentos sobrepostos (2011–2019). (Em R$ de 2019).

Ano	Gasto no ano (A)	Medicamentos sobrepostos^b^ (adquiridos no ano corrente e no ano anterior)				Gasto em medicamentos não sobrepostos^c^ (F) = (A) - (E)
		Preço (B)	Quantidade (C)	Alteração (D) = (B) + (C)	Gasto (E)	
2010	3.111.101.607	–	–	–	–	–
2011	3.964.405.181	−942.747.326	1.482.949.631	540.202.305	3.565.115.413	399.289.768
2012	2.730.880.595	−429.401.035	55.202.768	−374.198.267	2.224.139.542	506.741.053
2013	3.421.253.546	−409.206.701	262.862.794	−146.343.906	2.203.442.115	1.217.811.431
2014	3.374.752.748	−319.312.207	157.940.963	−161.371.244	2.403.345.441	971.407.307
2015	4.457.392.520	−152.411.230	−270.527.006	−422.938.236	2.143.408.273	2.313.984.247
2016	3.114.255.832	−1.065.511.265	−139.082.756	−1.204.594.021	2.834.934.548	279.321.284
2017	2.253.005.743	−317.851.837	−240.357.431	−558.209.268	1.848.589.843	404.415.900
2018	2.721.660.204	−214.323.069	503.440.831	289.117.762	1.866.013.403	855.646.801
2019	2.164.179.182	−104.657.061	−62.468.364	−167.125.425	1.509.499.956	654.679.226

Fonte: SIASG.

Elaboração própria.

a Medicamentos com DDD estabelecida.

b Não é possível separar a contribuição do resíduo decorrente de eventuais mudanças no padrão de uso dos medicamentos sobrepostos entre dois anos nesta análise, pois o gasto (E) se refere à despesa com a lista sobreposta nos dois períodos comparados, sendo esta lista diferente na série analisada, conforme se pode observar na coluna do número de fármacos sobrepostos da Tabela 1 deste artigo.

c O resíduo envolve as despesas decorrentes da aquisição de medicamentos não sobrepostos e as decorrentes de mudanças no padrão de uso dos medicamentos sobrepostos. Nesta coluna, apresentam-se os gastos no ano corrente relativos à contribuição dos medicamentos não sobrepostos.

Ademais, verifica-se na Tabela 3 que, em razão da redução dos preços, as maiores economias ocorreram em 2011 e 2016. Observam-se também os valores reduzidos em virtude da diminuição das quantidades em 2015 (R$ 270,53 milhões), 2016 (R$ 139,08 milhões), 2017 (R$ 240,36 milhões) e 2019 (R$ 62,47 milhões).

## DISCUSSÃO

Os resultados mostram grande impacto de alguns subgrupos terapêuticos sobre o gasto do MS. Em quase todos os anos, os imunossupressores foram o de maior despesa. Em análise que considerou todas as aquisições de medicamentos desse subgrupo pelos órgãos federais, identificou-se que o principal indutor do gasto foi a quantidade comprada no período de 2010 a 2015^19^. O aumento do gasto com imunossupressores tem relação com o aumento da realização de transplantes de órgãos e de medula no Brasil^20^, com a ampliação do acesso e com possível aumento das doenças autoimunes^21^. A queda do gasto identificada em 2019 em relação a 2018, considerando que o número de transplantes continuou crescendo nos últimos anos^20^, pode estar relacionada à redução da disponibilidade desses medicamentos para tratamento de doenças autoimunes e à redução dos preços desses produtos de forma geral.

Destaca-se o crescimento das despesas com antivirais para uso sistêmico utilizados no tratamento das hepatites B e C entre 2014 e 2016 e sua redução a partir de 2017. Nesse período, ocorreram mudanças no padrão de tratamento da hepatite C. Em 2015, foram incorporados três medicamentos: sofosbuvir, daclatasvir e simeprevir^22^. Dados de 2016 mostram que havia uma patente concedida para simeprevir e que, embora os outros dois medicamentos não estivessem protegidos por patentes, foram comercializados no Brasil de forma exclusiva por empresas farmacêuticas transnacionais^23^. A exclusividade de fornecimento em ambos os casos geralmente leva à oferta de produtos a preços elevados. Ainda em 2016, a Comissão Nacional de Incorporação de Tecnologias no SUS (Conitec) recomendou a exclusão dos medicamentos telaprevir e boceprevir^24^. Ademais, vários antivirais usados no tratamento da hepatite C foram objeto de Parcerias para o Desenvolvimento Produtivo e isso pode ter contribuído para a redução dos preços dos produtos ao longo do tempo^23^. Esses acontecimentos ajudam a explicar o movimento observado do gasto desse subgrupo terapêutico.

Quanto ao número de fármacos, houve ampliação do rol adquirido de forma centralizada pelo MS, como mostra a Tabela 1. Duas situações justificam esse incremento. A primeira é a pactuação entre os gestores do SUS para que o governo federal assuma a aquisição de medicamentos já incorporados ao sistema^4^. A segunda situação é a incorporação de medicamentos, que aumentou com a instituição da Conitec em 2011^25^ e que já entraram no SUS sob a responsabilidade de compra do MS^26^. Uma análise conjunta dos dados do Sistema de Informações Ambulatoriais (SIA) e das listas de medicamentos do CEAF revela que entre 2012 e 2018 o saldo de incorporações e desincorporações no grupo 1A foi de 17 fármacos e que a aquisição centralizada no MS ampliou em 26 fármacos (de 30 para 56)^27^.

É interessante notar ainda na Tabela 1 o percentual de sobreposição da lista de fármacos adquiridos entre dois anos consecutivos. Como em nenhum ano houve sobreposição de 100% e o número de fármacos adquiridos aumentou na maioria dos anos, tais fatos indicam centralização, incorporação e compra de item que já se encontrava sob a responsabilidade do MS que não foi adquirido no ano anterior. Além disso, como a sobreposição de fármacos ocorre em número menor no ano corrente em relação ao total de fármacos adquiridos no ano anterior, há evidências de que a aquisição não ocorre anualmente para diversos fármacos, pois é improvável que a maioria deles deixou de ser comprada por ter sido excluída do SUS^28^ e pela ocorrência de transferência de fármacos do grupo 1A para o grupo 1B^26^. Por exemplo, em 2019 foram adquiridos 25 fármacos que também foram comprados em 2018. Mas o total de fármacos adquiridos em 2018 foi de 36, logo, 11 fármacos comprados em 2018 não foram adquiridos em 2019.

O gasto em medicamentos não sobrepostos entre anos consecutivos foi particularmente alto em 2015 (R$ 2,3 bilhões), como mostra a Tabela 2. Tal fato está associado às decisões de centralização da compra e de incorporação de medicamentos entre 2014 e 2015. Após a decisão de incorporação, o SUS tem até 180 dias para disponibilizar os medicamentos em suas farmácias^29^. Outro elemento que chama a atenção nessa tabela é a redução do gasto no grupo dos medicamentos sobrepostos a partir de 2016 e no dos medicamentos não sobrepostos em 2016 em relação a 2015. No primeiro grupo, a variação das despesas entre 2016 e 2019 foi de -46,8% e, no segundo grupo, de -71,7% entre 2015 e 2019. Obviamente, a lista de medicamentos no segundo grupo difere a cada ano e a centralização ou incorporação de produtos mais ou menos caros pode influenciar o gasto. Porém, não se pode descartar que a redução seja resultante de restrições orçamentárias, agravadas com a aprovação da Emenda do Teto de Gastos em 2016 (EC 95). Entre 2016 e 2017, as despesas liquidadas do MS com o CEAF reduziram em 21,7%, passando de R$ 6,9 bilhões para R$ 5,4 bilhões a preços de 2017^7^.

Mais preocupante é a redução no primeiro grupo. Se entre 2010 e 2019 houve ampliação dos fármacos adquiridos pelo MS, seja por centralização ou por incorporação, era de se esperar aumento do gasto, a menos que os preços dos produtos reduzissem significativamente. Ademais, caso a quantidade de tratamentos diários por subgrupo terapêutico fosse constante, haveria indicação de que os medicamentos novos substituíram os mais antigos. De fato, os preços decresceram em todos os anos observados, como mostra a Figura 2, ampliando o poder de compra do MS em razão de sua capacidade de negociação com fornecedores exclusivos e pela escala de aquisição nas licitações. Contudo, a quantidade de tratamentos diários disponibilizada também reduziu de 2015 a 2017 e em 2019 em relação ao ano anterior. A Tabela 3 deixa claro o efeito da diminuição da quantidade sobre o gasto nesses anos.

Em síntese, o gasto do MS na aquisição de medicamentos do CEAF que têm DDD estabelecida oscilou no período de 2010 a 2019, com aumento significativo em meados dessa década. Entretanto, registrou queda de 30,4% na comparação entre 2019 em relação a 2010, apesar da centralização da compra e da incorporação de medicamentos terem suplantado a exclusão ou a transferência da responsabilidade da aquisição para as SES^27^. A queda dos preços dos tratamentos diários com fármacos sobrepostos em dois anos consecutivos contribuiu para essa redução em todo o período. Contudo, houve também redução da aquisição de tratamentos diários em anos recentes, com provável comprometimento da disponibilidade no SUS daqueles que tiveram a quantidade reduzida, considerando os relatos de falta de medicamentos do CEAF sob a responsabilidade do MS no SUS^30,31^. O aumento do resíduo em dois anos da série analisada foi o principal indutor do aumento do gasto nesses anos, o qual tem por causas principais a centralização da aquisição e a incorporação de medicamentos.

Este estudo apresenta, naturalmente, algumas limitações. A primeira é que a metodologia empregada impossibilita a análise dos medicamentos que não possuem DDD estabelecida e com isso 10,8% do gasto com o grupo 1A do CEAF não foram analisados. A segunda é que, uma vez que a base utilizada contempla somente dados de compras públicas, apenas foram estimados os efeitos dos indutores principais sobre o gasto. Com a utilização de dados complementares, por exemplo, os provenientes das Autorizações de Procedimentos de Alta Complexidade (APACs) e dos registros de medicamentos, é possível decompor os índices principais em outros índices, mensurando a contribuição de indutores como a aquisição de genéricos, volume e extensão das prescrições, entre outros, que impactam os indutores principais^2,32^. Por fim, a terceira limitação envolve a informação sobre a quantidade comprada e consequentemente sobre o valor total, pois, na metodologia aplicada neste estudo, o gasto com cada medicamento é calculado a partir da multiplicação do preço unitário e da quantidade negociada ou prevista na aquisição, constante do SIASG. O gasto anual é a soma do gasto com os medicamentos adquiridos no ano. Ainda que no Painel de Compras do Governo Federal encontrem-se informações sobre a execução orçamentário-financeira do MS por contrato, não se tem a informação da despesa empenhada, liquidada e paga por item de medicamento com a respectiva quantidade. Dessa forma, trabalhou-se com a quantidade prevista na aquisição.

Entretanto, apesar dessas limitações, o estudo aporta contribuição significativa para o entendimento dos principais fatores que impactam a maior parte da despesa do MS no grupo 1A CEAF. A análise dos indutores do gasto em medicamentos se mostra uma ferramenta útil para a gestão da assistência farmacêutica, pois produz informação que possibilita uma compreensão mais ampla sobre o comportamento de variáveis que impactam as despesas, podendo orientar a adoção de medidas visando a garantir o acesso da população aos medicamentos e o uso racional dos recursos financeiros.
